# Potent effect of the MDM2 inhibitor AMG232 on suppression of glioblastoma stem cells

**DOI:** 10.1038/s41419-018-0825-1

**Published:** 2018-07-18

**Authors:** Nam-Gu Her, Jeong-Woo Oh, Yun Jeong Oh, Suji Han, Hee Jin Cho, Yeri Lee, Gyu Ha Ryu, Do-Hyun Nam

**Affiliations:** 1 Institute for Refractory Cancer Research, Samsung Medical Center, Seoul, 06351 Korea; 2 Department of Health Sciences & Technology, Samsung Advanced Institute for Health Science & Technology, Sungkyunkwan University, Seoul, 06351 Korea; 3 Office of R&D Strategy & Planning, Samsung Medical Center, Seoul, 06351 Korea; 4 Department of Neurosurgery, Samsung Medical Center, Sungkyunkwan University, Seoul, 06351 Korea

## Abstract

Testing new ways to identify untapped opportunities for glioblastoma therapies remains highly significant. Amplification and overexpression of MDM2 gene is frequent in glioblastoma and disrupting the MDM2−p53 interaction is a promising strategy to treat the cancer. RG7112 is the first-in class inhibitor and recently discovered AMG232 is the most potent MDM2 inhibitor known to date. Here, we compared the effects of these two clinical MDM2 inhibitors in six glioblastoma cell lines and ten patient-derived glioblastoma stem cells. Targeted sequencing of the *TP53*, *MDM2* genes and whole transcriptome analysis were conducted to verify genetic status associated with sensitivity and resistance to the drugs. Although *TP53* wild-type glioblastoma cell lines are similarly sensitive to AMG232 and RG7112, we found that four *TP53* wild-type out of ten patient-derived glioblastoma cells are much more sensitive to AMG232 than RG7112 (average IC_50_ of 76 nM vs. 720 nM). Among these, 464T stem cells containing *MDM2* gene amplification were most sensitive to AMG232 with IC_50_ of 5.3 nM. Moreover, AMG232 exhibited higher selectivity against p53 wild-type cells over p53 mutant stem cells compared to RG7112 (average selectivity of 512-fold vs. 16.5-fold). Importantly, we also found that AMG232 is highly efficacious in three-dimensional (3D) tumor spheroids growth and effectively inhibits the stemness-related factors, Nestin and ZEB1. Our data provide new evidence that glioblastoma stem cells have high susceptibility to AMG232 suggesting the potential clinical implications of MDM2 inhibition for glioblastoma treatment. These will facilitate additional preclinical and clinical studies evaluating MDM2 inhibitors in glioblastoma and direct further efforts towards developing better MDM2-targeted therapeutics.

## Introduction

p53 is the most frequently mutated tumor suppressor gene whose somatic alterations are found in approximately 50% of all human cancers^1^. It regulates a wide array of cellular processes such as DNA repair, growth arrest, and apoptosis depending on the cellular context^2^. The mechanisms by which p53 plays its tumor suppressor roles have been well studied. The level of p53 is kept very low under normal conditions mostly by a post-translational mechanism involving the ubiquitin-proteasome system^3^. The oncogene MDM2 serves as an E3 ubiquitin ligase that destabilizes and negatively regulates the p53 protein^4^. In response to diverse cellular inputs such as oncogenic and genotoxic stresses, the interaction between MDM2 and p53 is disrupted, the p53 level increases and therefore it activates or represses the target genes important for protecting cells from malignant transformation^5^. Although half of all tumors retain wild-type p53, its activity is largely attenuated as a result of MDM2 overexpression or other mechanisms^6^.

Targeting MDM2 to reactivate p53 function is a promising strategy to treat cancers. Hence, intensive efforts to develop small-molecule inhibitors of MDM2‑p53 interaction have been made over the last decade^7–9^. Nutlins are preclinical molecules first identified through a chemical library screening^10^ and their analog RG7112 was the first-in class MDM2 inhibitor^11^. Several other MDM2 inhibitors such as RG7388, MI77301, CGM097, MK8242, and AMG232 entered clinical trials^12–16^. Among these, AMG232 is the most potent MDM2 inhibitor described to date^17^.

Glioblastoma is the most prevalent and lethal primary brain tumor of which median survival is only ~14 months^18^. Treatment of glioblastoma currently relies on surgical tumor resection and radiochemotherapy that provide only limited benefit to patients^19,20^. Although new approaches have been explored, only few has proven effective in treating glioblastoma so far^21^. Thus, testing new strategies to improve survival of glioblastoma patients remains highly significant. Amplification and overexpression of *MDM2* gene is observed in 8–10% of glioblastoma^22^ and a recent study demonstrated that the first-in class drug RG7112 has a preclinical efficacy in glioblastoma^23^. These suggest that targeting MDM2 should be considered as one of treatment options for glioblastoma.

Here, we used RG7112 and AMG232 to test the effect of MDM2 inhibitors in glioblastoma cells. We measured cell number and biomarker immunofluorescence to evaluate RG7112 and AMG232 across six glioblastoma cell lines and ten patient-derived glioblastoma stem cells. We found that AMG232 is more effective and selective in p53 wild-type patient-derived glioblastoma stem cells. This effect was more evident in 3D tumor spheroids growth supporting the prominent role of AMG232 in inhibition of glioblastoma stemness. Our data provide a new insight into possibility of p53 reactivation strategies in inhibition of glioblastoma stem cells and treating glioblastoma.

## Results

### Evaluation of the MDM2 inhibitors RG7112 and AMG232 in glioblastoma cell lines

**Fig. 1:**
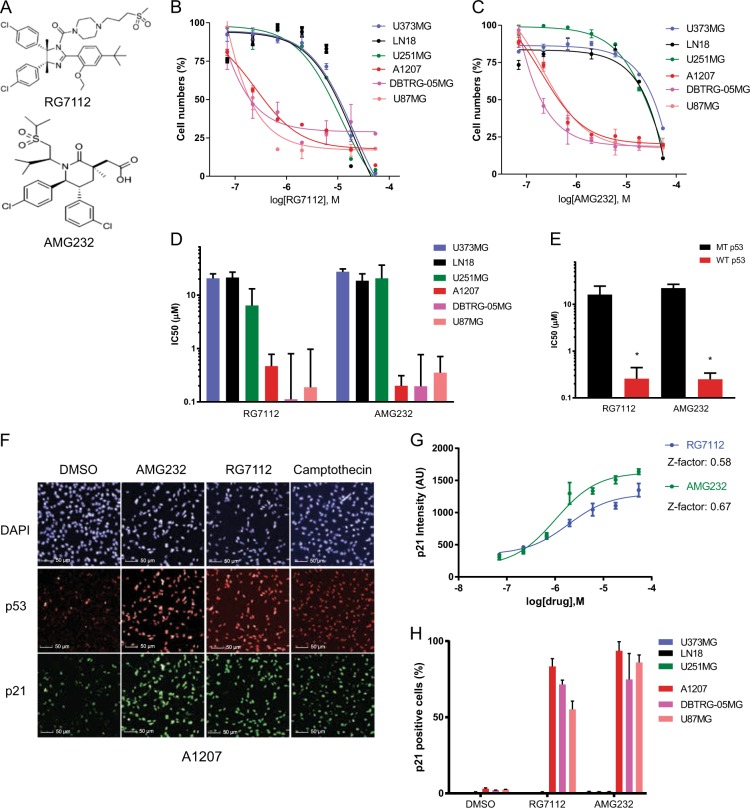
The effect of MDM2 inhibitors, RG7112 and AMG232 in glioblastoma cell lines. **a** Chemical structures of RG7112 and AMG232. **b**, **c** Cell numbers of U373MG, LN18, U251MG, A1207, DBTRG-05MG, and U87MG cells treated with different concentrations (0.07–54 μM) of RG7112 or AMG232 for 72 h were evaluated by quantifying image-based cell counting. Nonlinear regression analyses of dose−response curves are shown (*n* = 3). Error bars represent standard deviation (SD). **d** The IC_50_ values obtained from dose−response curves are shown. Each bars represent mean (*n* = 3) and 95% confidence interval (CI). **e** IC_50_ values of *TP53* mutant cell lines (*n* = 3) and *TP53* wild-type cell lines (*n* = 3) to RG7112 and AMG232 are shown. Data represent the mean and SD error (*p < 0.01). **f** An image-based immunofluorescence assay for p21 (green), p53 (red) and DAPI (blue) in A1207 cells treated with DMSO, RG7112 (2 μM), AMG232 (2 μM), and Camptothecin (10 μM) for 72 h are shown. **g** p21 fluorescence intensity measurements for A1207 cells treated with increasing concentrations of RG7112 or AMG232 for 72 h are shown. The Z-factor for 18 μM RG7112 and 18 μM AMG232 in A1207 cells are indicated. **h** The mean percentage of p21-positive cells (*n* = 3, SD) for six glioblastoma cell lines treated with DMSO, RG7112 (2 μM), AMG232 (2 μM) for 72 h are shown

In order to compare the effect of RG7112 and AMG232 (Fig. 1a) in glioblastoma cell lines, we tested the sensitivity of previously known *TP53* mutant cell lines (U373MG, LN18, and U251MG) and *TP53* wild-type cell lines (A1207, DBTRG-05MG, and U87MG)^24–26^ to the drugs. We used a cell-based screening platform for high content analysis that concurrently measures both cell numbers and biomarker immunofluorescence in 384-well plate format to quantitatively evaluate the drug responses. Analysis of cell numbers using the assay with increasing concentrations of RG7112 and AMG232 are shown in Fig. 1b, c. Half-maximal growth-inhibitory concentration (IC_50_) values of RG7112 in cell lines are 20.67 μM (U373MG), 21.33 μM (LN18), 6.41 μM (U251MG), 0.47 μM (A1207), 0.11 μM (DBTRG-05MG), and 0.18 μM (U87MG) (Fig. 1d). IC_50_ values of AMG232 are 27.36 μM (U373MG), 18.54 μM (LN18), 20.70 μM (U251MG), 0.20 μM (A1207), 0.19 μM (DBTRG-05MG), and 0.35 μM (U87MG) (Fig. 1d). As expected, *TP53* wild-type cell lines (A1207, DBTRG-05MG, and U87MG) were sensitive to both MDM2 inhibitors, while *TP53* mutant cell lines (U373MG, LN18, and U251MG) were generally insensitive to the drugs (Fig. 1e). It is notable, however, that no significant differences were observed in the sensitivity of *TP53* wild-type glioblastoma cell lines between RG7112 and AMG232 despite the reported higher biochemical potency of AMG232 in the literature^11,16^. We also observed that immunofluorescence readouts for p53 and its target p21 are increased by the MDM2 inhibitors and known DNA-damaging agent Camptothecin^27^ in *TP53* wild-type A1207 cells (Fig. 1f and Supplementary Fig. 1a). Dose−response result of p21 assay testing RG7112 and AMG232 on A1207 cells showed concentration-dependent changes in p21 levels and provided sufficient quality for using p21 as a predictive biomarker of MDM2 inhibition (0.58 and 0.67 of Z-factors for RG7112 and AMG232, respectively) (Fig. 1g). Unlike A1207 cells, *TP53* mutant cell lines U373MG and LN18 cells did not show an increase in p21 levels to the MDM2 inhibitors (Supplementary Fig. 1b). The mean percentage of p21-positive cells upon RG7112 or AMG232 treatment in three *TP53* mutant and three *TP53* wild-type cell lines were ~3% and >50%, respectively and overall p21 responsiveness correlated with the sensitivity in all cell lines we studied (Fig. 1h). Additionally, we validated the reliability and consistency of our image-based cell counting analysis by comparing with traditional ATP-based cell viability assay and image-based cell viability assay (Supplementary Fig. 2a and 2b). Collectively, our high content analysis data demonstrate that *TP53* wild-type glioblastoma cell lines are similarly sensitive to RG7112 and AMG232, while *TP53* mutant glioblastoma cell lines are insensitive to the drugs.

### Reactivation of p53 is critical for the sensitivity of glioblastoma cells to the MDM2 inhibitors

**Fig. 2:**
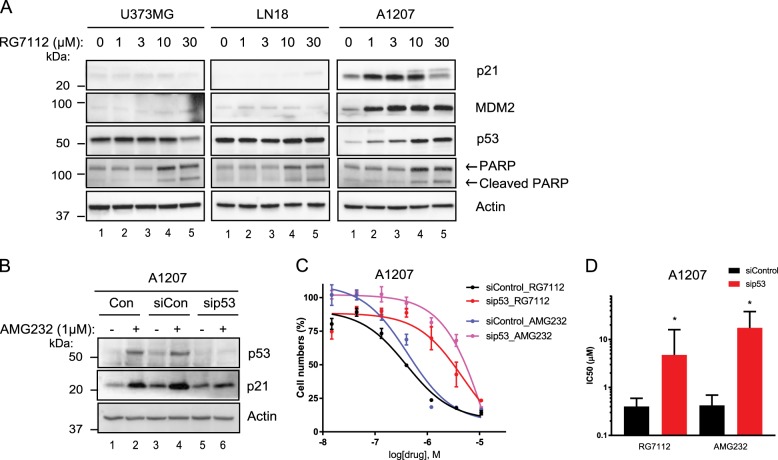
Reactivation of p53 is associated with the sensitivity of glioblastoma cells to the MDM2 inhibitors. **a** U138MG, LN18, and A1207 cells were treated with RG7112 for 12 h at the indicated concentrations. p21, MDM2, p53, and PARP levels were examined by immunoblotting. Actin was used as a loading control. **b** A1207 cells were transfected with control or p53 siRNA for 48 h prior to treatment with DMSO or 1 μM AMG232 for 12 h. The levels of p53 and p21 were examined by immunoblotting. Actin was used as a loading control. **c** A1207 cells transfected with control or p53 siRNA for 48 h were treated with different concentrations (0.014–10.8 μM) of RG7112 or AMG232 for 72 h were evaluated by quantifying image-based cell counting. Nonlinear regression analyses of dose−response curves are shown (*n* = 3, SD error). **d** The graph represents IC_50_ values comparing control and p53-depleted A1207 cells for RG7112 and AMG232. Data show mean and SD error (*p < 0.01)

To confirm the relationship between wild-type p53 and the MDM2 inhibitor sensitivity as observed in Fig. 1, we examined the levels of p53 and its target genes in response to RG7112 at the molecular levels. As shown in Fig. 2a, the steady-state levels of p53, p21, and MDM2 are increased by RG7112 in a concentration-dependent manner in *TP53* wild-type A1207 cells but not in *TP53* mutant U373MG and LN18 cells. Intriguingly, there were no significant differences in the cleaved PARP levels among these cells at 10 and 30 μM RG7112. We observed similar sub-G1 populations and Caspase 3/7 activities in A1207 cells and LN18 cells treated with 10 μM or higher concentrations of RG7112 suggesting that *TP53*-independent cytotoxicity could occur at high doses of the MDM2 inhibitors consistent with the result in Fig. 1b (Supplementary Fig. 3a and 3b). Next, we explored whether p53 depletion affect the response of cells to the MDM2 inhibitors. We transfected p53 siRNA into A1207 cells to silence endogenous p53 (Fig. 2b) and analyzed cell numbers of control and p53-depleted cells to the MDM2 inhibitors (Fig. 2c). Knockdown of p53 in A1207 cells resulted in >11.9-fold decreased sensitivity measured by IC_50_ to the MDM2 inhibitors (Fig. 2d). In addition, we found p53-null HCT116 cells showed >75.9-fold decreased sensitivity measured by IC_50_ to the MDM2 inhibitors compared to parental HCT116 cells (Supplementary Fig. 4a and 4b). Collectively, these data validated that reactivation of p53 is highly important for the sensitivity of cells to the MDM2 inhibitors.

### Evaluation of the MDM2 inhibitors RG7112 and AMG232 in patient-derived glioblastoma stem cells

**Fig. 3:**
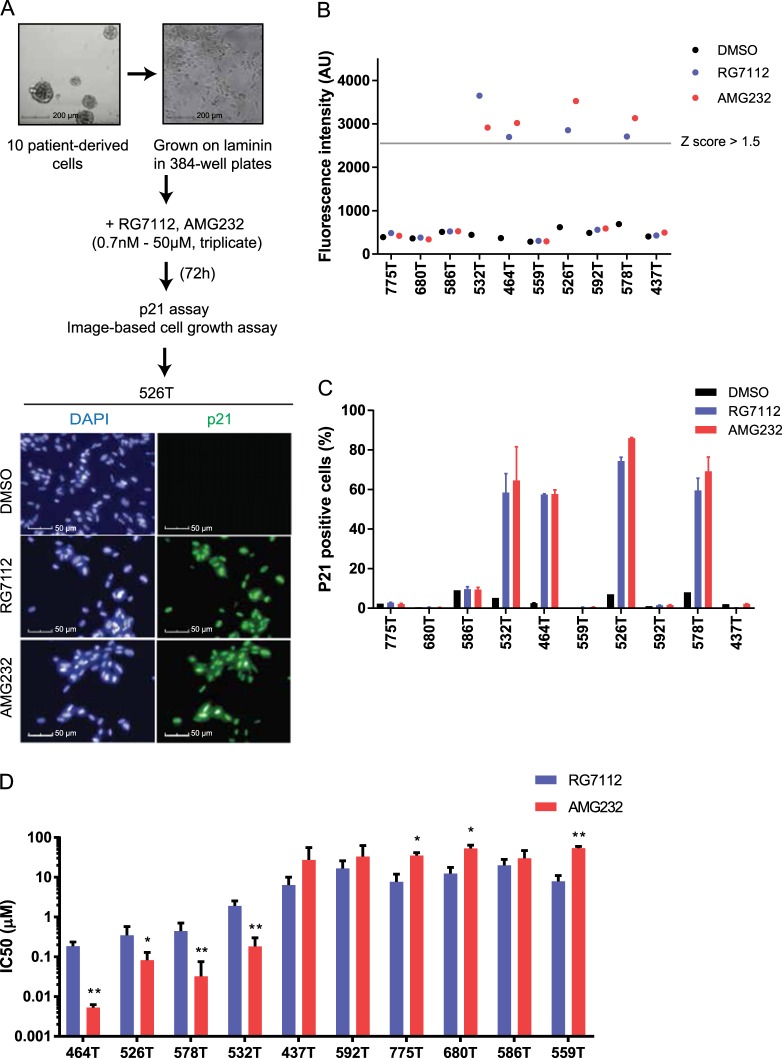
The effect of MDM2 inhibitors, RG7112 and AMG232 in patient-derived glioblastoma stem cells. **a** An experiment design of high content analysis for patient-derived glioblastoma stem cells are represented. Ten patient-derived glioblastoma stem cells were isolated and grown as neurosphere as shown in upper left panel. Stem cells were cultured in laminin-coated 384-well plates (upper right panel) were treated with RG7112 and AMG232 (0.7 nM−50 μM) for 72 h and were analyzed by automated microscopy for image-based cell counting and p21 immunofluorescence assay. Representative images of p21 (green) and DAPI (blue) in 526T stem cells treated with DMSO, RG7112 (1.8 μM), AMG232 (1.8 μM) for 72 h are shown in lower panel. **b** p21 fluorescence intensity measurements for DMSO, RG7112 (1.8 μM), and AMG232 (1.8 μM)-treated cells (*n* = 3). The gray line indicates the threshold (Z-score = 1.5). **c** The mean percentage of p21-positive cells for ten patient-derived glioblastoma stem cells treated with DMSO, RG7112 (1.8 μM), and AMG232 (1.8 μM) for 72 h are shown. Data represent mean (*n* = 3) and SD error. **d** IC_50_ values comparing RG7112 and AMG232 in ten patient-derived glioblastoma stem cells obtained from dose−response curves are shown (*n* = 3, CI error, *p < 0.05, **p < 0.01). Nonlinear regression analysis for these data are shown in Supplementary Figure 6

Because we could not detect differences in the effect of RG7112 and AMG232 in our cell lines study, we questioned whether AMG232 could better sensitize glioblastoma than RG7112. As long-term cultured cell lines cannot recapitulate the molecular features of original glioblastoma, patient-derived glioblastoma stem cells cultured without serum could be physiologically more relevant models for in vitro studies^28^. Therefore, we sought to test RG7112 and AMG232 in patient-derived glioblastoma stem cells. Ten glioblastoma patients-derived cells expanded as neurosphere were grown on laminin, treated with increasing concentrations of RG7112 and AMG232 for high content analysis and resulting images for 526T stem cells are shown Fig. 3a. p21 levels were markedly increased by RG7112 and AMG232 in four (464T, 526T, 532T and 578T) out of ten stem cells suggesting that these stem cells might be sensitive to the drugs (Supplementary Fig. 5). As shown in Fig. 3b, c, these four stem cells showed greater p21 fold changes (Z-score > 1.5) and p21-positive populations ( > 50%) upon the MDM2 inhibitors treatment, whereas no significant differences were found in six stem cells (437T, 559T, 586T, 592T, 680T, and 775T). We observed that cells showing an increase in the p21 levels were highly sensitive to AMG232 (IC_50_, 5.3–183 nM) and RG7112 (IC_50_, 0.2–1.9 μM)_,_ while the p21-unresponsive cells were insensitive (IC_50_ of AMG232, 27–54 μM; IC_50_ of RG7112, 6.4–20 μM) (Fig. 3d, See Supplementary Fig. 6a and 6b for the detailed data). Particularly, 464T cells were extremely sensitive to AMG232 with IC_50_ of 5.3 nM (Supplementary Fig. 6c). These data suggest that the AMG232 is more effective than RG7112 in a subset of glioblastoma stem cells. Our observations also suggest that MDM2 inhibitor-insensitive six stem cells are more resistant to AMG232 than RG7112.

### Genome analysis verified the sensitivity predictors of the MDM2 inhibitors

**Fig. 4:**
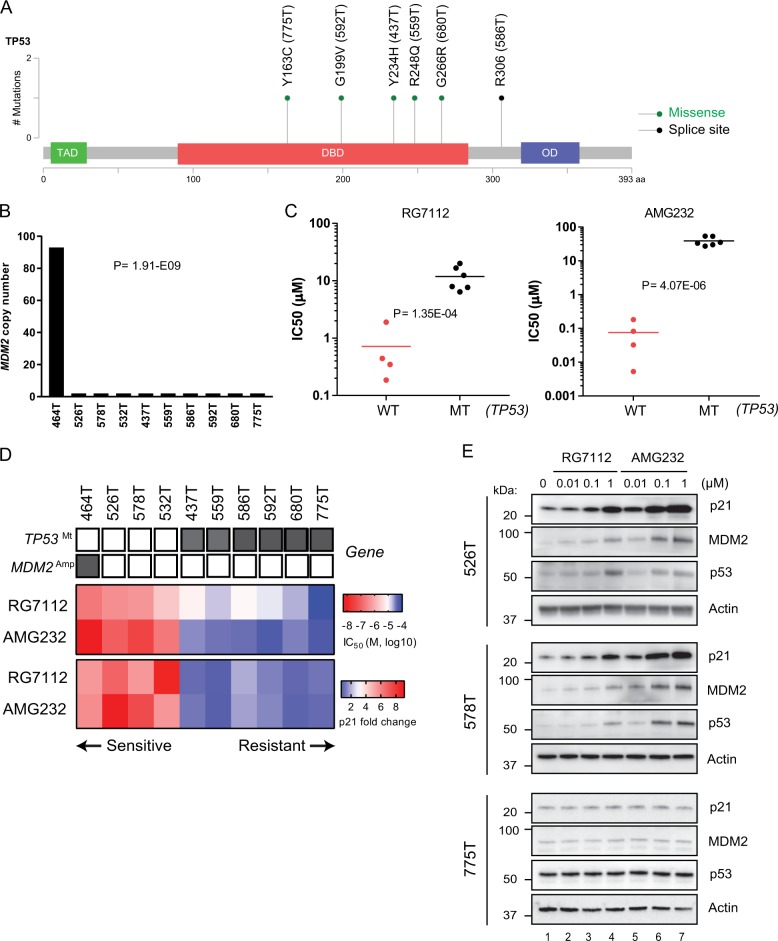
Integrated genome and high content analyses verify genetic status of patient-derived glioblastoma stem cells associated with high sensitivity to AMG232. **a** Location of *TP53* gene mutation in six patient-derived glioblastoma stem cells. Green circles represent missense mutations and a black circle indicates a splice-site mutation. The amino acid change is indicated above each circle and the name of patient-derived glioblastoma stem cells is bracketed. **b** The graph shows *MDM2* gene copy numbers in ten patient-derived glioblastoma stem cells. *P* value is indicated in the graph. **c** Scatter plots showing IC_50_ values of patient-derived glioblastoma stem cells to RG7112 and AMG232. The stem cells are classified according to *TP53* mutation status. *P* value is indicated in the graph. **d** A map of gene-high content analysis association. Genetic status of *TP53*, *MDM2*, and IC_50_, p21 fold-changes of the MDM2 inhibitors in ten patient-derived glioblastoma cells are summarized. Color scales with IC_50_ and Z-score values are indicated. **e** 526T, 578T, and 775T stem cells were treated with RG7112 and AMG232 for 72 h at the indicated concentrations. p21, MDM2, and p53 levels were evaluated by immunoblotting. Actin was used as a loading control

We next analyzed targeted sequencing of the *TP53* and *MDM2* genes and whole *RNA* sequencing of patient-derived glioblastoma stem cells used in this study to identify key genetic status related to the drug sensitivity. As expected, point mutations in the *TP53* gene including five missense and one splice site were found in all six insensitive patient-derived stem cells while no mutation was identified in four sensitive stem cells (Fig. 4a). In addition, overexpression of the *MDM2* transcript as a result of gene amplification was found in 464T stem cells which were shown to be the most sensitive to the drugs (Fig. 4b and Supplementary Fig. 7). These results further verified that *TP53*-wild-type stem cells are associated with the sensitivity to AMG232 and RG7112 (Fig. 4c). The mean IC_50_ value of AMG232 and RG7112 in *TP53* wild-type stem cells were 76 and 720 nM, respectively. In *TP53* mutant stem cells, however, the mean IC_50_ value of AMG232 and RG7112 were 38.9 and 11.9 μM, respectively. These data indicate that AMG232 is 512-fold selective and RG7112 is 16.5-fold selective in *TP53* wild-type over *TP53* mutant stem cells. We displayed a heat map summarizing the genetic status of TP53, MDM2, and p21 assay (fold change) and IC_50_ values of ten stem cells with AMG232 and RG7112 in Fig. 4d. Moreover, we examined the p53, p21, and MDM2 levels and observed their increases by the drugs in *TP53* wild-type stem cells 526T and 578T cells but not in *TP53* mutant 775T stem cells (Fig. 4e). Collectively, these data demonstrate that *TP53* wild-type patient-derived glioblastoma stem cells are more sensitive and selectively respond to AMG232 than RG7112.

### Inhibition of the glioblastoma stemness by AMG232

**Fig. 5:**
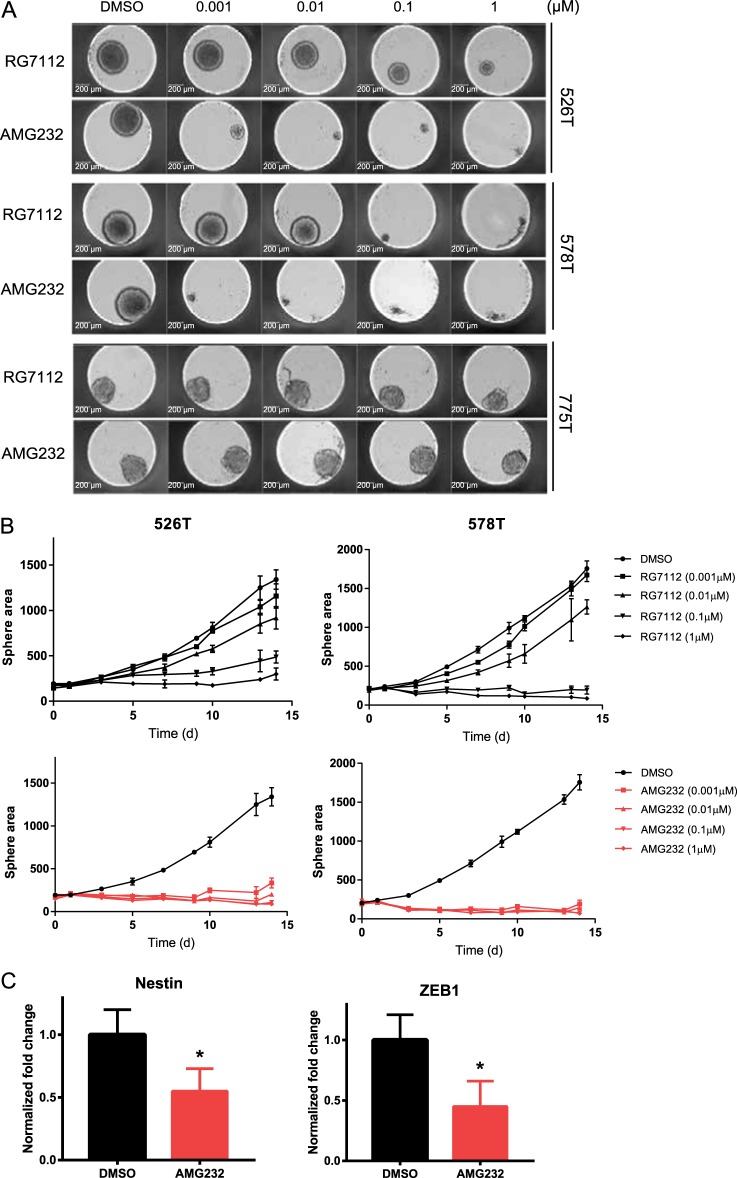
AMG232 suppresses Nestin, ZEB1 and stemness of patient-derived glioblastoma cells. **a** Three-dimensional (3D) tumor spheroids of 526T, 578T, and 775T patient-derived glioblastoma cells were treated with indicated concentrations of RG7112 and AMG232. Representative images show the response of 3D spheroids to RG7112 and AMG232 at day 14. **b** The sphere images were taken at 2-day intervals up to 14 days and the sizes were measured and analyzed. Data represent the mean (*n* = 3) and SD error. **c** 578T cells were treated with 0.1 μM AMG232 for 72 h and analyzed by immunoblotting (see Supplementary Figure 9). The steady-state levels of Nestin and ZEB1 relative to Actin were quantified. Data show the mean and error (*n* = 2, SD, *p < 0.05)

Our observations suggest that glioblastoma stem cells are highly susceptible to the effect of AMG232. To further confirm these in physiologically more meaningful system, we grew 3D tumor spheroids produced from patient-derived glioblastoma stem cells with different concentrations of AMG232 and RG7112 up to 2 weeks. As shown in Fig. 5a, 1 nM of AMG232 was sufficient to completely inhibit 3D tumor growth of *TP53* wild-type 526T and 578T stem cells, while 100~1000-fold higher (0.1–1 μM) RG7112 was required to exert the similar effect. On the other hand, 3D tumor growth of *TP53*-mutant 775T cells were unaffected to the maximum concentration (1 μM) of both drugs. The in vitro 3D tumor response to the MDM2 inhibitors was successfully tracked for 2 weeks showing that AMG232 has strong efficacy and selectivity in *TP53* wild-type patient-derived glioblastoma stem cells (Fig. 5b and Supplementary Fig. 8). To study potential mechanisms of the AMG232 sensitivity in tumor spheroids growth, we examined if AMG232 regulates the glioblastoma stemness-related factors. Downregulation of the steady-state levels of Nestin and ZEB1 was observed supporting that AMG232 modulates stemness regulators linked to maintenance of the glioblastoma 3D growth (Fig. 5c and Supplementary Fig. 9). Together, these data highlight the potent effect of AMG232 in suppression of glioblastoma stem cells.

## Discussion

As the cellular responses to targeted therapeutics are affected by the genetic characteristics in each tumor, there have been substantial efforts towards identifying specific genetic alterations associated with drug sensitivity^29^. One of the strongest gene−drug associations identified through previous high throughput screens was *TP53*-MDM2 inhibitor^30^. This is consistent with enthusiasm for development of MDM2-targeted small molecules thus far owing to its capacity to selectively suppress *TP53* wild-type tumors. Given that MDM2 inhibitors have emerged as a promising therapeutic tool and are under clinical studies now, evaluation of them in clinically relevant samples with diverse genetic backgrounds represents an informative insight. In the present study, we tested one of the most potent MDM2 inhibitors, AMG232 in glioblastoma cell lines and patient-derived glioblastoma stem cells for the first time. The informed mutation status and expression profile of key genetic factors in patient-derived cells demonstrated the association between AMG232 and wild-type TP53 that were stronger than those observed in studies which evaluated other MDM2 inhibitors^23^.

Glioblastoma, among the most malignant tumor, is regarded as incurable and fatal with fewer than 5% of patients surviving more than 5 years. Surgical resection followed by radiation and chemotherapy with Temozolomide provides only transient benefits to patients and lose effectiveness over time^31^. Since attempts to achieve advances in treating glioblastoma have been disappointing over the past 30 years, new treatment options are urgently required^32^. Therefore, addressing how cancer cells respond to molecularly targeting agents that are potentially applicable to treat glioblastoma should facilitate better understanding of the limitations and possibilities for the disease treatment. Recently, Verreault et al. described the effect of the first-in class MDM2 inhibitor RG7112 in patient-derived glioblastoma cell lines in vitro and in vivo^23^. Their preclinical study has shed light on potential clinical implication of using MDM2 inhibition strategies to fight glioblastoma. A concern that reactivating wild-type p53 function causes widespread apoptosis in normal cells necessitates the use of highly specific and potent MDM2 inhibitors in clinical testing^33^. Our data suggest that AMG232 is 9.5-fold more effective than RG7112 in *TP53* wild-type glioblastoma cells. Of note, MDM2-amplified stem cells (464T) were 35-fold more sensitive to AMG232 with IC_50_ of 5.3 nM (RG7112 IC_50_, 185 nM). Moreover, the effect of AMG232 on long-term cultured 3D tumor spheroids growth of glioblastoma stem cells was 100~1000-fold stronger than RG7112 presenting compelling evidence for its cellular effect. Regarding the selectivity, AMG232 is shown to be effective in *TP53* wild-type glioblastoma cells 512-fold and >1000-fold higher in adherent culture and tumor spheroids, respectively. These data imply that AMG232 inhibition of the p53−MDM2 interaction is more specific and highly regulated than RG7112. Notably, AMG232 has recently entered clinical trials for patients with recurrent or newly diagnosed glioblastoma (clinicaltrials.gov/ct2/show/NCT03107780) suggesting that AMG232 is likely to penetrate blood−brain barrier and potentially applicable to glioblastoma patients.

Traditional high throughput screen measures single parameter cell viability but image-based high content analysis can examine more diverse aspects of the drug responses involving biomarker and phenotype-based readouts providing better reliability of the assay^34^. In order to obtain efficient and accurate cellular effects of AMG232, we used high content analysis that simultaneously monitors both p21 immunofluorescence and cell numbers. The screen statistics and additional verification of the assay established sufficient confidence to utilize the assay in prediction and selection of glioblastoma patients who are likely to respond to the MDM2 inhibitors in the future. Likewise, image-based high content analysis has recently emerged as an efficient approach for personalized medicine; it may contribute to empowering precision oncology^35,36^.

Glioblastoma stem cells are a rare population within a tumor and are defined by its self-renewing and tumor initiating abilities^37^. It has been proposed that glioblastoma stem cells enhance tumorigenic properties and confer therapeutic resistance; therefore isolation and application to in vitro drug screen are critical to comprehend how they behave upon drug treatment. Also, use of glioblastoma stem cells in preclinical studies has been promoted since long-term cultured cancer cell lines in serum containing medium cannot accurately reflect the genetic profiles of the original patient tumors^28^. Here, we used glioblastoma stem cells of early passages derived from the patients. While the effect of AMG232 in glioblastoma cell lines was similar to RG7112, its effect in glioblastoma stem cells was much stronger. These show that glioblastoma stem cells are more vulnerable to AMG232 suggesting that its effect depends on the cellular context. Glioblastoma stem cells rely on multiple key stemness-related proteins such as Nestin, CD133, SOX2, Olig2, BMI1, and ZEB1^38,39^. We found AMG232 suppresses Nestin and ZEB1 indicating the inhibitor leads to inhibition of the glioblastoma stemness. This is consistent with previous findings that p53 represses Nestin transcription and ZEB1 levels^40,41^. These will stimulate further investigations into the molecular basis of glioblastoma susceptibility to p53 reactivation.

Collectively, this study provides an important new insight into the effects of MDM2 inhibition and suppression of glioblastoma stemness using AMG232. Our work will facilitate additional explorations of MDM2 inhibitors for the potential clinical testing and contribute to the development of improved MDM2 inhibitors.

## Materials and Methods

### Cell lines and patient-derived glioblastoma stem cells

Three *TP53* mutant glioblastoma cell lines (U373MG, LN18, and U251MG) and three *TP53* wild-type glioblastoma cell lines (A1207, DBTRG-05MG, and U87MG) were either purchased from the American Type Culture Collection (ATCC) or kindly provided by Prof. Shin-Hyuk Kang at Korea University, Seoul, Korea. Cells were grown in DMEM with 10% fetal bovine serum and 1% antibiotics. Tumor specimens were obtained from adult glioblastoma patients who underwent surgery at Samsung Medical Center and informed consent was received. Samsung Medical Center Institutional Review Board approved this study (IRB file #201512092). Patient-derived glioblastoma cells were grown as previously described^28^. Briefly, surgical samples were enzymatically dissociated into single cells and cultured in neurobasal-A media (Gibco) supplemented with N2, B27, 1X l-Glutamin (Gibco), 1% penicillin/streptomycin (Gibco), human recombinant bFGF and EGF.

### Reagents and antibodies

AMG232 and RG7112 were purchased from APExBIO Technology. Anti-p21 (#2947), anti-PARP (#9542), and anti-ZEB1 (#3396) were purchased from Cell Signaling, anti-p53 (sc-6243), anti-MDM2 (sc-813), and anti-Nestin (sc-23927) were purchased from Santa Cruz Biotechnology and anti-beta-actin (ab8227) was purchased from Abcam. Alexa Fluor 488 anti-rabbit secondary antibody (A11008) was purchased from Thermo Fisher Scientific. Laminin (L2020) was purchased from Sigma and Hoechest33342 (H3570) was purchased from Life Technologies.

### High content analysis, cell viability, and apoptosis assay

Two thousand cells were seeded in CellCarrier 384-well plate (Perkin Elmer) for high content analysis. In case of patient-derived stem cells, plates were precoated with 1:100 diluted laminin in PBS as previously described^42^. Cell were treated with drugs after 24 h, fixed with 4% paraformaldehyde and blocked with PBS containing 1% BSA, 0.3% Triton X-100 for 1 h at 72 h post-treatment. Then cells were incubated with anti-p21 antibody followed by Alexa Fluor 488 secondary antibody and Hoechest33342. Cell images were taken using Operetta (Perkin Elmer) and analyzed by Harmony High Content Analysis software (Perkin Elmer) according to the manufacturer’s guidelines. For measuring cell numbers, nucleus of viable cells was segmented based on nuclear size and shape, counted for each well and total cells were normalized for percent cell numbers. IC_50_ values were determined using Prism 7 software (Graphpad) as previously described^43^. For p21 immunofluorescence analysis, Alexa fluor 488 images were taken and mean intensities per cell were analyzed. For analyses of percent p21-positive cells, fluorescence intensities cut-off for exclusion of background signal was 400 (Arbitrary Unit, AU). Z-factor and Z-scores were calculated as previously described^44^. For cell viability assay measuring cellular ATP, ATPlite 1step and Envision microplate reader (Perkin Elmer) were used. For apoptosis assay, the Caspase3/7 activity was evaluated using Cell event Caspase3/7 detection reagent (Thermo Fisher) and fluorescence intensities per cell were analyzed. For image-based cell viability assay, Caspase 3/7-positive fluorescence cells (cut-off: 300–1000 (AU) depending on cell types) were regarded as dead cells and excluded from total cells for counting viable cells.

### siRNA transfection

Transfection of siRNAs was performed using Oligofectamine (Invitrogen) according to the manufacturer’s instructions. siRNAs against control and p53 are kindly provided by Prof. Sung-Gil Chi at Korea University, Seoul, Korea.

### Cell cycle analysis

Glioblastoma cell line A1207 and LN18 cells were fixed with 70% ethanol. The cells were resuspended in 2 ml of PBS containing 100 μg/ml RNase and 50 mg/ml propidium iodide. Cell cycle analysis was conducted on a FACScan flow cytometer (Becton Dickinson), and the analysis of cell cycle profile was performed using MultiCycle software (Phoenix Flow Systems).

### Immunoblot assay

Cells were lysed in complete lysis-M buffer (Roche Applied Science) and clarified by centrifugation. After the concentration was measured using Bio-Rad Protein assay kit (Bio-Rad), proteins were mixed with laemmli buffer and boiled. Then equal amount (20 μg) of proteins were fractionated by SDS-PAGE and transferred to PVDF membrane using the iBlot 2 system (Life Technologies). The membrane was blocked in 2% non-fat dry milk for 30 min, incubated with primary antibodies at 4 °C overnight and then with secondary antibodies for 1 h at room temperature. SuperSignal West Pico Plus (Thermo Fisher Scientific) was used to detect antibody binding.

### Three-dimensional cell growth assay

Three-dimensional spheroids were generated and cultured in the InSphero Gravity TRAP ULA 96-well plates (Perkin Elmer) according to the manufacturer’s instruction. One thousand cells per well were seeded for initial spheroids formation and images were taken at 2-day intervals up to 14 days using Operetta (Perkin Elmer). Sphere areas were quantified using the Image Studio software (LI-COR).

### Targeted gene sequencing

Targeted-exome sequencing called GliomaSCAN^TM^, which covers only glioma-associated genes, was performed for GBM patient-derived cells. The resulting sequenced reads were mapped to human genome (hg19) with the Burrows−Wheeler Aligner (BWA)^45^. To detect single nucleotide variants and small indels, MuTect and SomaticIndelDetector (GATK) were used and Variant Effect Predictor (VEP) annotated the called mutations^46–49^. For copy number estimation, ONCOCNV was used^50^.

### RNA sequencing

With the Illumina TruSeq RNA sample Prep Kit and Illumina HiSeq2500, RNA-seq was conducted for GBM patient-derived cells. The sequenced reads (100 nt) were trimmed to 30 nt from the 5′-end and then mapped to hg19 using GSNAP^51^. DEGseq was used to determine gene expression levels as RPKM for human RefSeq genes^52^.

## Electronic supplementary material

Supplementary Figures

## References

[1] Olivier, M., Hollstein, M. & Hainaut, P. TP53 mutations in human cancers: origins, consequences, and clinical use. *Cold Spring Harb. Perspect. Biol*. **2**, a001008 (2010). 10.1101/cshperspect.a001008 PMC2827900 20182602

[2] Brady CA, Attardi LD (2010). p53 at a glance. J. Cell Sci..

[3] Yang Y, Li CC, Weissman AM (2004). Regulating the p53 system through ubiquitination. Oncogene.

[4] Moll UM, Petrenko O (2003). The MDM2−p53 interaction. Mol. Cancer Res..

[5] Mello SS, Attardi LD (2017). Deciphering p53 signaling in tumor suppression. Curr. Opin. Cell Biol..

[6] Oliner, J. D., Saiki, A. Y. & Caenepeel, S. The role of MDM2 amplification and overexpression in tumorigenesis. *Cold Spring Harb. Perspect. Med*. **6**, a026336 (2016). 10.1101/cshperspect.a026336 PMC4888815 27194168

[7] Zhang B, Golding BT, Hardcastle IR (2015). Small-molecule MDM2-p53 inhibitors: recent advances. Future Med. Chem..

[8] Zhao Y, Aguilar A, Bernard D, Wang S (2015). Small-molecule inhibitors of the MDM2-p53 protein−protein interaction (MDM2 Inhibitors) in clinical trials for cancer treatment. J. Med. Chem..

[9] Wang, S., Zhao, Y., Aguilar, A., Bernard, D. & Yang, C. Y. Targeting the MDM2-p53 protein−protein interaction for new cancer therapy: progress and challenges. *Cold Spring Harb. Perspect. Med*. **7**, a026245 (2017). 10.1101/cshperspect.a026245 PMC5411684 28270530

[10] Vassilev LT (2004). In vivo activation of the p53 pathway by small-molecule antagonists of MDM2. Science (New York, N.Y.).

[11] Vu B (2013). Discovery of RG7112: A small-molecule MDM2 inhibitor in clinical development. ACS Med. Chem. Lett..

[12] Ding Q (2013). Discovery of RG7388, a potent and selective p53-MDM2 inhibitor in clinical development. J. Med. Chem..

[13] Wang S (2014). SAR405838: an optimized inhibitor of MDM2-p53 interaction that induces complete and durable tumor regression. Cancer Res..

[14] Holzer P (2015). Discovery of a dihydroisoquinolinone derivative (NVP-CGM097): a highly potent and selective MDM2 inhibitor undergoing phase 1 clinical trials in p53wt tumors. J. Med. Chem..

[15] Ravandi F (2016). A phase I trial of the human double minute 2 inhibitor (MK-8242) in patients with refractory/recurrent acute myelogenous leukemia (AML). Leuk. Res..

[16] Sun D (2014). Discovery of AMG 232, a potent, selective, and orally bioavailable MDM2-p53 inhibitor in clinical development. J. Med. Chem..

[17] Canon J (2015). The MDM2 inhibitor AMG 232 demonstrates robust antitumor efficacy and potentiates the activity of p53-inducing cytotoxic agents. Mol. Cancer Ther..

[18] Khosla D (2016). Concurrent therapy to enhance radiotherapeutic outcomes in glioblastoma. Ann. Transl. Med..

[19] Lacroix M (2001). A multivariate analysis of 416 patients with glioblastoma multiforme: prognosis, extent of resection, and survival. J. Neurosurg..

[20] Gallego O (2015). Nonsurgical treatment of recurrent glioblastoma. Curr. Oncol..

[21] Mrugala MM (2013). Advances and challenges in the treatment of glioblastoma: a clinician’s perspective. Discov. Med..

[22] Reifenberger G, Liu L, Ichimura K, Schmidt EE, Collins VP (1993). Amplification and overexpression of the MDM2 gene in a subset of human malignant gliomas without p53 mutations. Cancer Res..

[23] Verreault M (2016). Preclinical efficacy of the MDM2 inhibitor RG7112 in MDM2-amplified and TP53 wild-type glioblastomas. Clin. Cancer Res..

[24] Van Meir EG (1994). Analysis of the p53 gene and its expression in human glioblastoma cells. Cancer Res..

[25] Barone TA (2017). Anticancer drug candidate CBL0137, which inhibits histone chaperone FACT, is efficacious in preclinical orthotopic models of temozolomide-responsive and -resistant glioblastoma. Neuro. Oncol..

[26] Kruse CA (1992). Characterization of a continuous human glioma cell line DBTRG-05MG: growth kinetics, karyotype, receptor expression, and tumor suppressor gene analyses. Vitr. Cell. Dev. Biol..

[27] Yoshida A, Ueda T, Wano Y, Nakamura T (1993). DNA damage and cell killing by camptothecin and its derivative in human leukemia HL-60 cells. Jpn. J. Cancer Res.: Gann.

[28] Lee J (2006). Tumor stem cells derived from glioblastomas cultured in bFGF and EGF more closely mirror the phenotype and genotype of primary tumors than do serum-cultured cell lines. Cancer Cell..

[29] Barretina J (2012). The Cancer Cell Line Encyclopedia enables predictive modelling of anticancer drug sensitivity. Nature.

[30] Garnett MJ (2012). Systematic identification of genomic markers of drug sensitivity in cancer cells. Nature.

[31] Ramirez YP, Weatherbee JL, Wheelhouse RT, Ross AH (2013). Glioblastoma multiforme therapy and mechanisms of resistance. Pharmaceuticals (Basel, Switzerland).

[32] Lee JK, Nam DH, Lee J (2016). Repurposing antipsychotics as glioblastoma therapeutics: Potentials and challenges. Oncol. Lett..

[33] Khoo KH, Verma CS, Lane DP (2014). Drugging the p53 pathway: understanding the route to clinical efficacy. Nat. Rev. Drug. Discov..

[34] Boutros M, Heigwer F, Laufer C (2015). Microscopy-based high-content screening. Cell.

[35] Chia S (2017). Phenotype-driven precision oncology as a guide for clinical decisions one patient at a time. Nat. Commun..

[36] Sant, G. R., Knopf, K. B. & Albala, D. M. Live-single-cell phenotypic cancer biomarkers-future role in precision oncology? *NPJ. Precis. Oncol*. **1**, 21 (2017) 10.1038/s41698-017-0025-y PMC5871838 29872705

[37] Lathia JD, Mack SC, Mulkearns-Hubert EE, Valentim CL, Rich JN (2015). Cancer stem cells in glioblastoma. Genes Dev..

[38] Bradshaw A (2016). Cancer stem cell hierarchy in glioblastoma multiforme. Front. Surg..

[39] Siebzehnrubl FA (2013). The ZEB1 pathway links glioblastoma initiation, invasion and chemoresistance. EMBO Mol. Med..

[40] Tschaharganeh DF (2014). p53-dependent Nestin regulation links tumor suppression to cellular plasticity in liver cancer. Cell.

[41] Kim T (2011). p53 regulates epithelial−mesenchymal transition through microRNAs targeting ZEB1 and ZEB2. J. Exp. Med..

[42] Pollard SM (2009). Glioma stem cell lines expanded in adherent culture have tumor-specific phenotypes and are suitable for chemical and genetic screens. Cell Stem Cell.

[43] Her NG (2016). p97 composition changes caused by allosteric inhibition are suppressed by an on-target mechanism that increases the enzyme’s ATPase activity. Cell Chem. Biol..

[44] Bray, M. A., & Carpenter, A. Imaging platform, B.I.o.M.I.T. & Harvard advanced assay development guidelines for image-based high content screening and analysis. In *Assay Guidance Manual* (eds. Sittampalam, G. S. et al.) (Eli Lilly & Company and the National Center for Advancing Translational Sciences, Bethesda, MD, 2004). 23469374

[45] Li H, Durbin R (2009). Fast and accurate short read alignment with Burrows-Wheeler transform. Bioinformatics (Oxford, England).

[46] Cibulskis K (2013). Sensitive detection of somatic point mutations in impure and heterogeneous cancer samples. Nat. Biotechnol..

[47] DePristo MA (2011). A framework for variation discovery and genotyping using next-generation DNA sequencing data. Nat. Genet..

[48] McKenna A (2010). The Genome Analysis Toolkit: a MapReduce framework for analyzing next-generation DNA sequencing data. Genome Res..

[49] McLaren W (2010). Deriving the consequences of genomic variants with the Ensembl API and SNP Effect Predictor. Bioinformatics (Oxford, England).

[50] Boeva V (2014). Multi-factor data normalization enables the detection of copy number aberrations in amplicon sequencing data. Bioinformatics (Oxford, England).

[51] Wu TD, Nacu S (2010). Fast and SNP-tolerant detection of complex variants and splicing in short reads. Bioinformatics (Oxford, England).

[52] Wang L, Feng Z, Wang X, Wang X, Zhang X (2010). DEGseq: an R package for identifying differentially expressed genes from RNA-seq data. Bioinformatics (Oxford, England).

