# Supplementary material for: Potent effect of the MDM2 inhibitor AMG232 on suppression of glioblastoma stem cells
Source: Cell Death Dis. 2018 Jul 18;9(8):792. doi: 10.1038/s41419-018-0825-1 (PMC6052082; doi:10.1038/s41419-018-0825-1)

Supplementary Figure Legends

Supplementary Fig. 1 Biomarker immunofluorescence assays obtained through high content analysis.

a p21 and p53 fluorescence intensity measurements for A1207 cells treated with or without 10μM Camptothecin for 72 hours are shown (N=2 and SD error, * $p < 0.01$). **b** Representative images of for p21 (green), p53 (red) and DAPI (blue) for U373MG, LN18 and A1207 cells treated with increasing concentrations of RG7112 or AMG232 for 72 hours are shown.

Supplementary Fig. 2 Comparison of ATP-based cell viability assay, high content analysis (HCA)-based cell viability assay and HCA-based cell number analysis.

a U373MG, LN18, A1207 and U87MG cells were treated with different concentrations (0.07 - 54μM) AMG232 for 72 hours, and were evaluated by ATP-based cell viability assay and HCA-based cell viability assay. IC_{50} values obtained from dose response curves from ATP-based cell viability and HCA-based cell viability assays are compared with HCA-based cell number analysis. (N=3 and CI error). **b** The scatter plots comparing IC_{50} values between the assay methodologies for measuring drug responses are shown. Pearson correlation coefficient was calculated based on IC_{50} values and indicated in the plots.

Supplementary Fig. 3 High concentrations of RG7112 cause TP53-independent cytotoxicity of glioblastoma cells. a LN18 and A1207 cells were treated with DMSO, 10 and 30μM RG7112 for 24 hours and sub-G1 fraction was analyzed by flow cytometer and resulting data are shown in graph (right panel). **b** U373MG, LN18 and A1207 cells were treated with different concentrations (0.07 - 54μM) RG7112 for 72 hours, and Image-based Caspase3/7 assay was performed. Fluorescence intensity per cell are analyzed and shown (N=3, SD error).

Supplementary Fig. 4 Decreased sensitivity of *TP53*-null HCT116 cells to the MDM2 inhibitors. a HCT116 (p53+/+) and HCT116 (p53-/-) cells were treated with different concentrations (0.07 - 54μM) of RG7112 or AMG232 for 72 hours. Cell numbers were analyzed and nonlinear regression analyses of dose response curves are shown (n=3, SD error). **b** The graph represents IC_{50} values comparing HCT116 (p53+/+) and HCT116 (p53-/-) cells for RG7112 and AMG232. Data show mean and SD error (* $p < 0.01$)

Supplementary Fig. 5 An increase in the p21 level is a sensitivity predictor of the MDM2 inhibitors. An immunofluorescence assay for p21 in 437T, 775T, 680T, 559T, 532T, 578T and 464T cells upon RG7112 and AMG232 treatment.

Supplementary Fig. 6 Dose response curves of RG7112 and AMG232 in 10 patient-derived glioblastoma cells. a Nonlinear regression analyses of dose response curves for RG7112 (70nM - 50μM) in 9 cells are shown (n=3, SD) **b** Nonlinear regression analyses of dose response curves for AMG232 (70nM - 50μM) in 9 cells are shown (n=3, SD) **c** Nonlinear regression analyses of dose response curves for RG7112 and AMG232 (0.7nM – 500nM) in 464T cells are shown (n=3, SD).

Supplementary Fig. 7 Increased expression of *MDM2* mRNA in 464T cells. The RPKM values of *MDM2* mRNA in 10 patient-derived glioblastoma cells.

Supplementary Fig. 8 Resistance of the 775T spheroid to the MDM2 inhibitors. The sphere growth of 775T cells in response to RG7112 and AMG232 (1nM - 1μM) for 14 days were analyzed. Data represent the mean (n=3) and error (SD).

Supplementary Fig. 9 The steady-state levels of Nestin, ZEB1 and N-Cadherin are reduced by AMG232. 578T cells treated with 0.1μM AMG232 for 72 hours were examined

by immunoblot analysis. The levels of Nestin, ZEB1, N-Cadherin, p53 and p21 were shown and Actin was used as a loading control.

Supplementary Fig. 10 The full scan for all the western blot images in this study.

Supplementary Figure 1

A

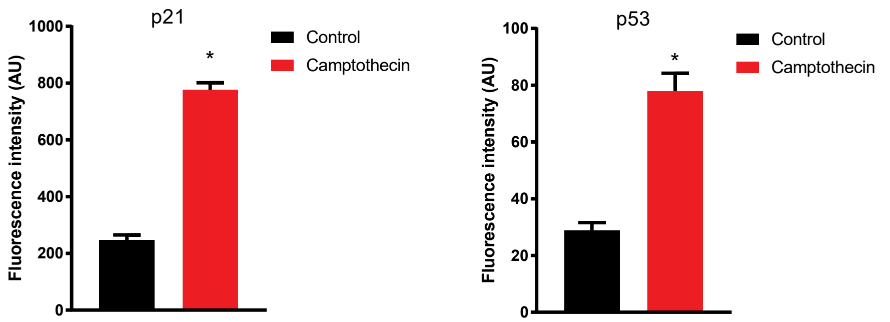

B

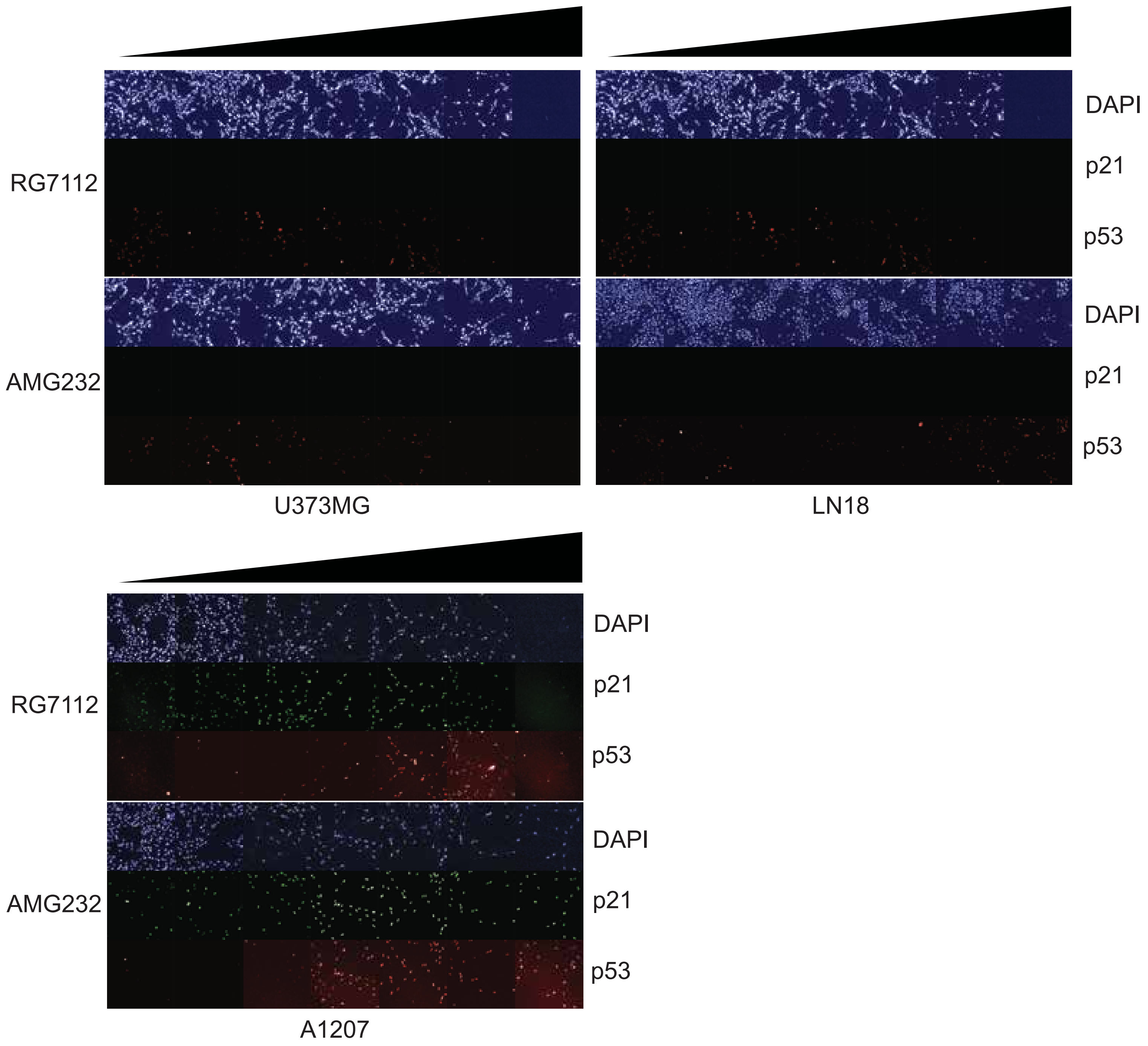

Supplementary Figure 2

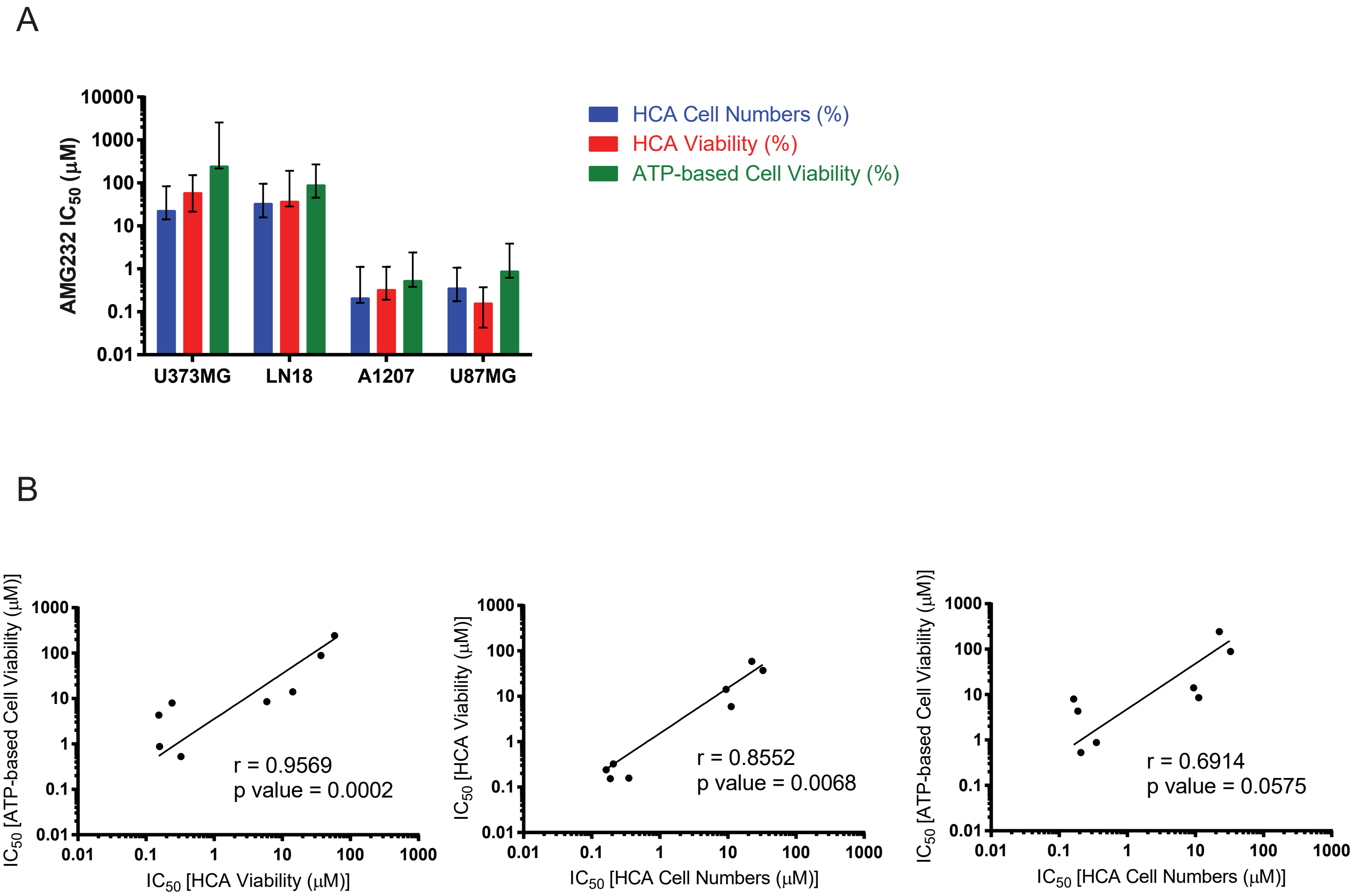

Supplementary Figure 3

A

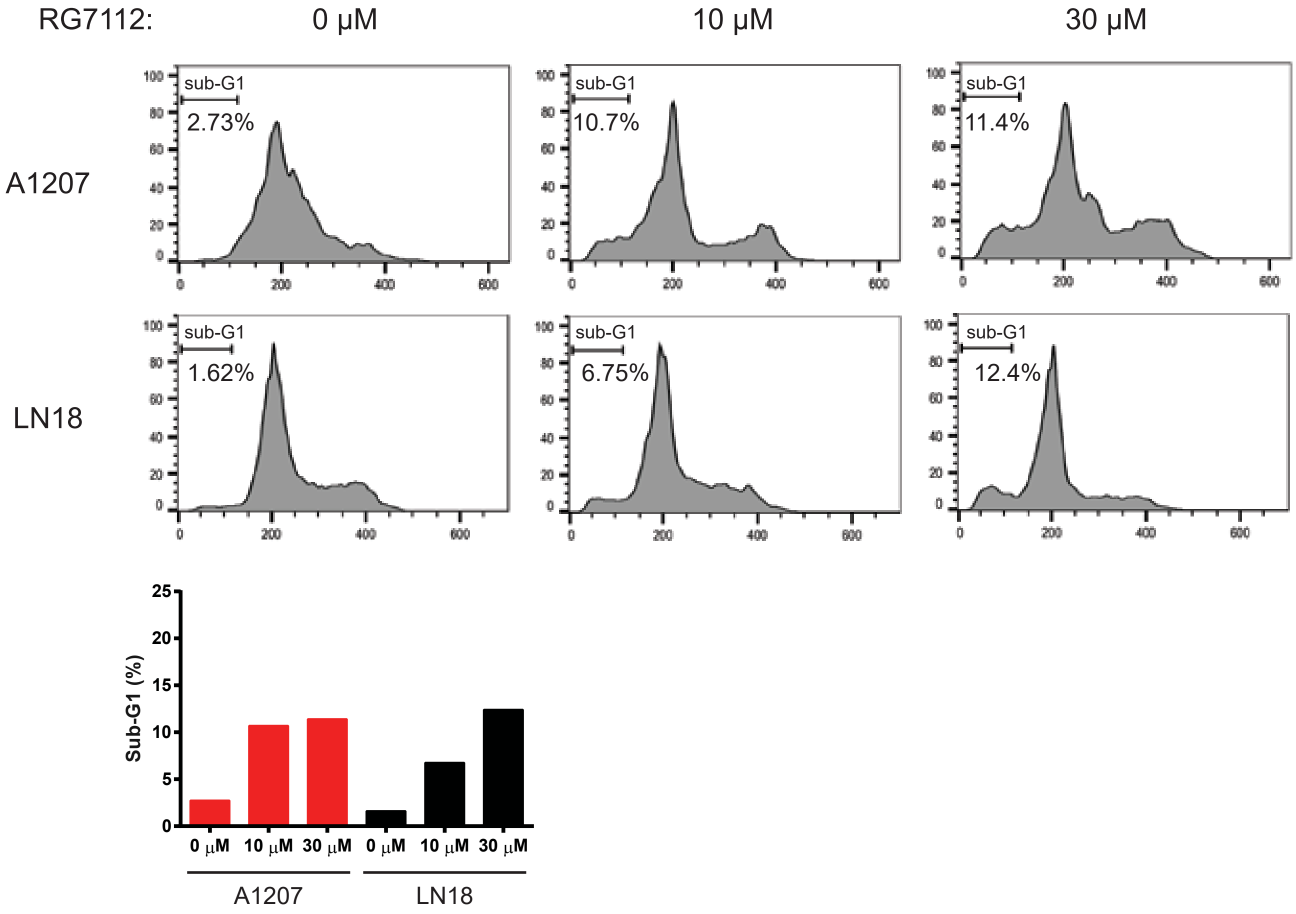

B

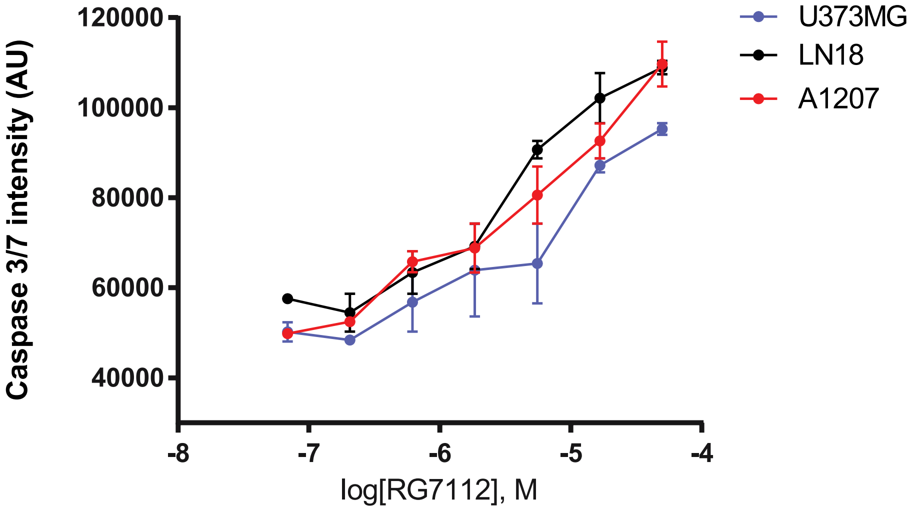

Supplementary Figure 4

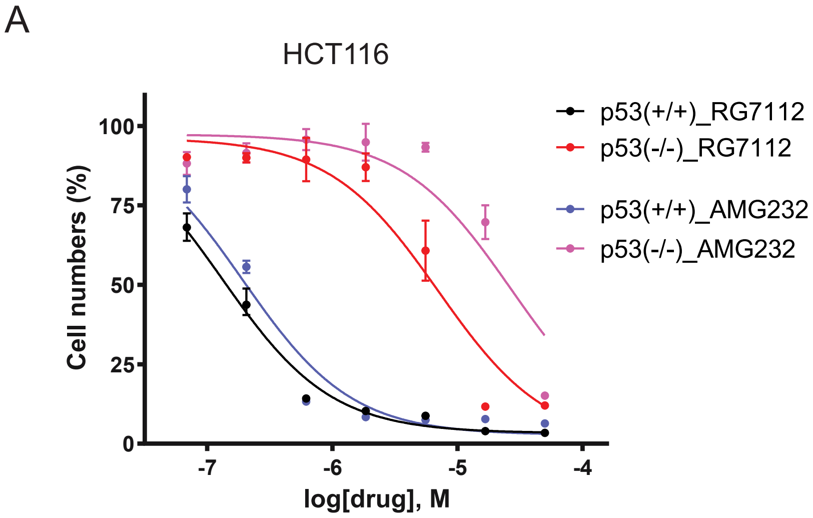

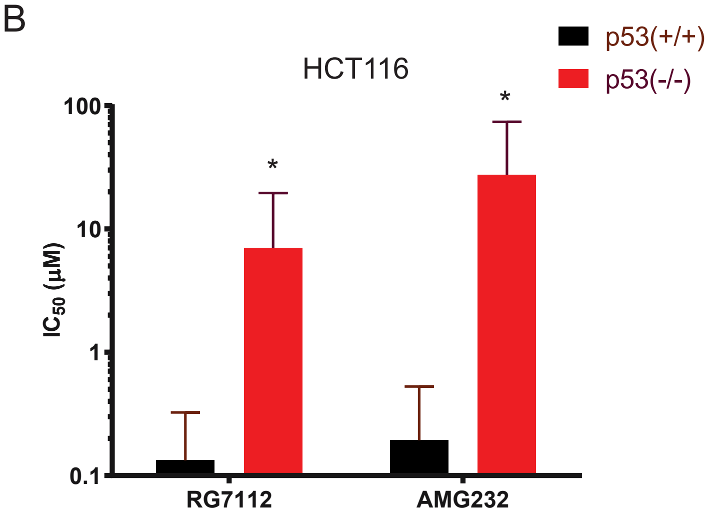

Supplementary Figure 5

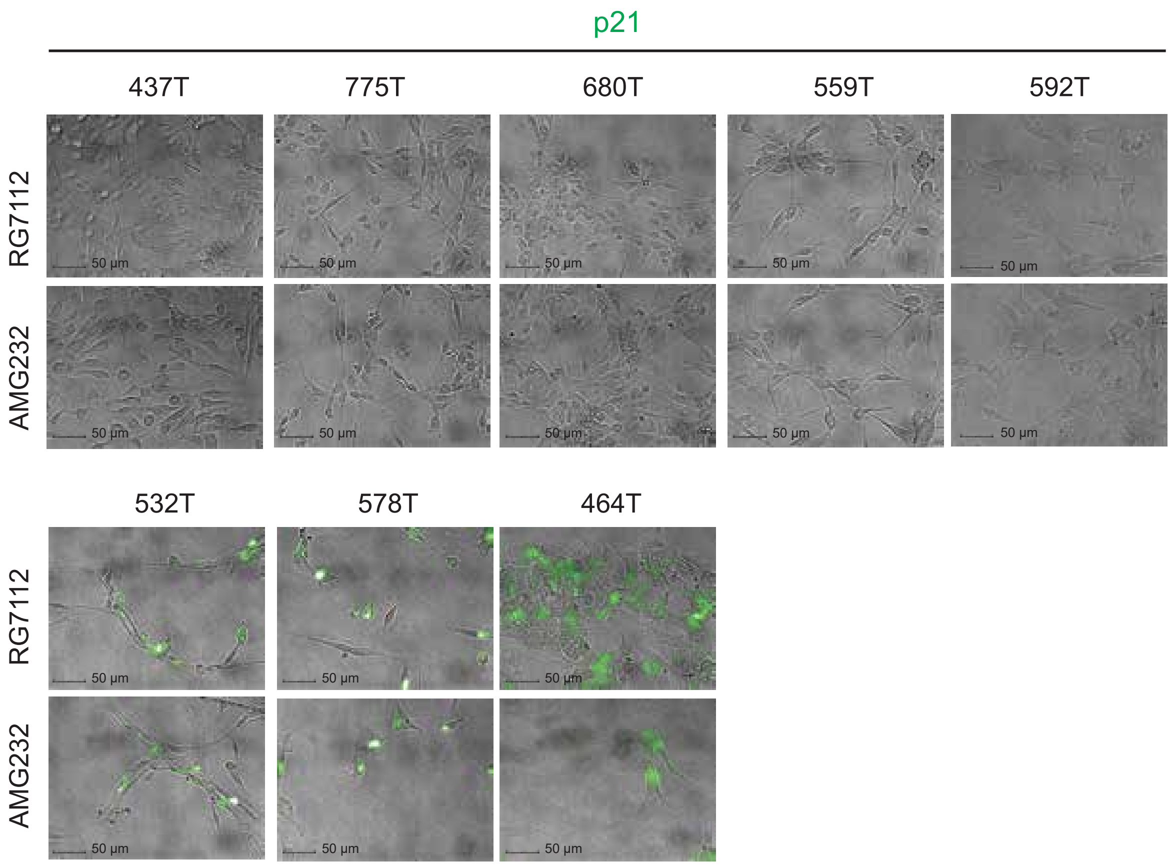

Supplementary Figure 6

a

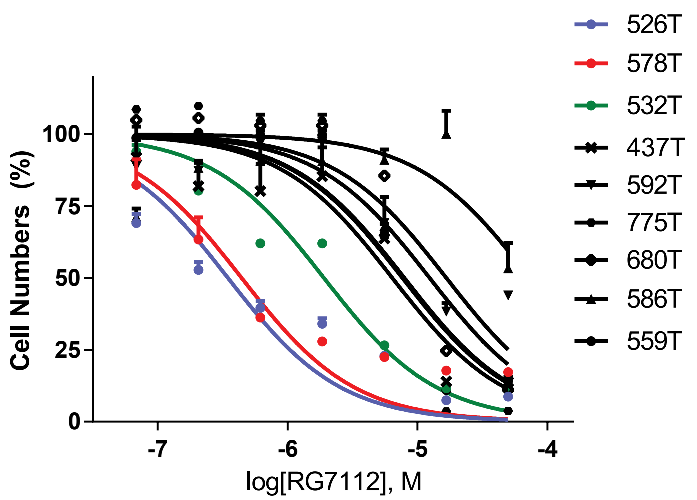

b

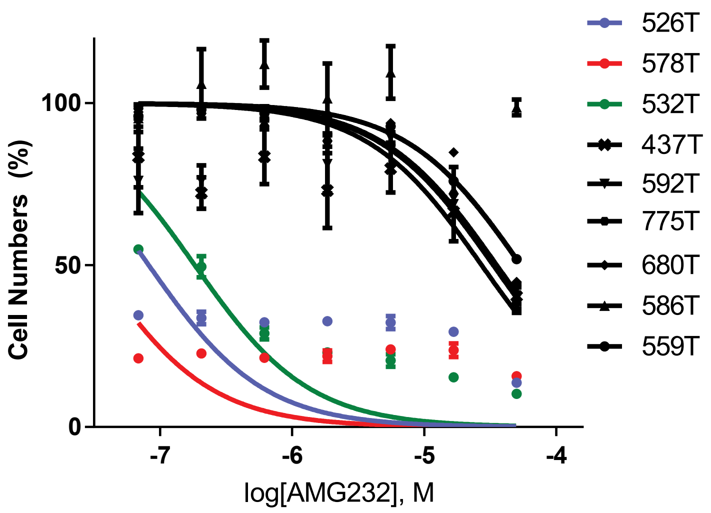

c

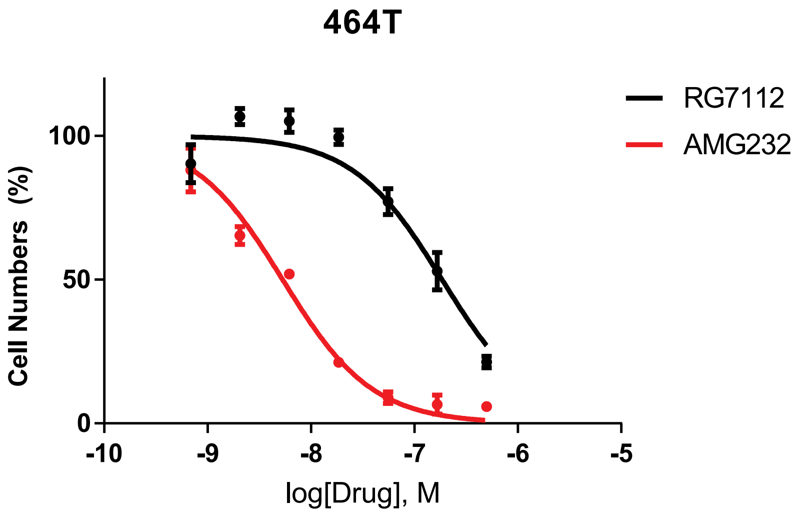

Supplementary Figure 7

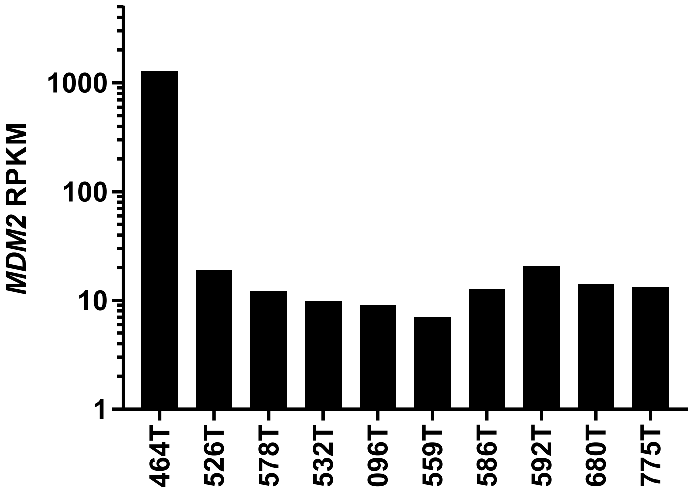

Supplementary Figure 8

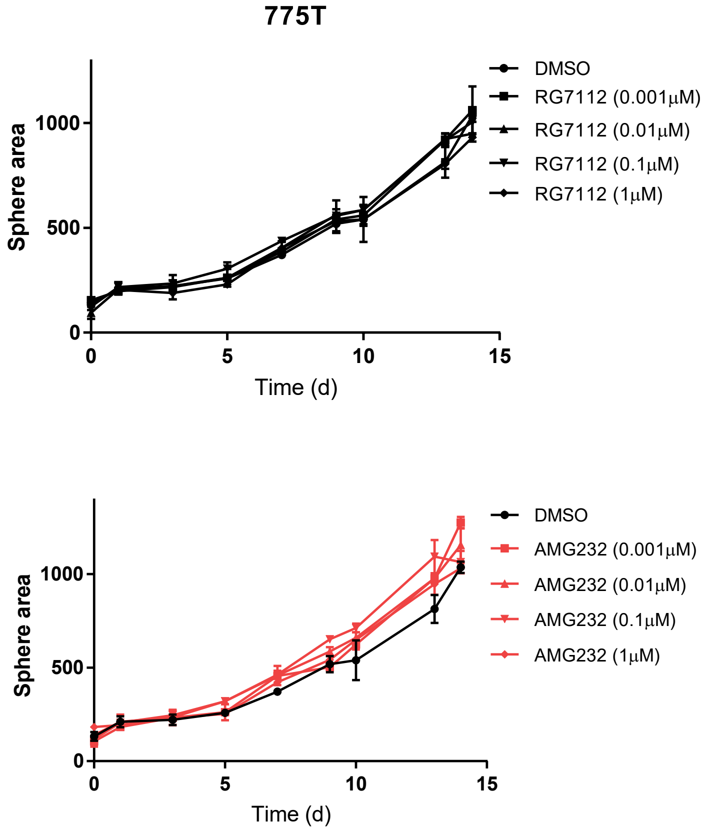

Supplementary Figure 9

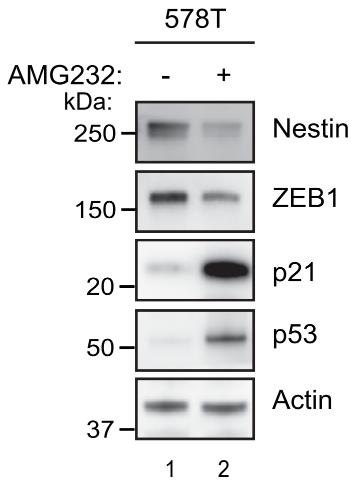

Supplementary Figure 10

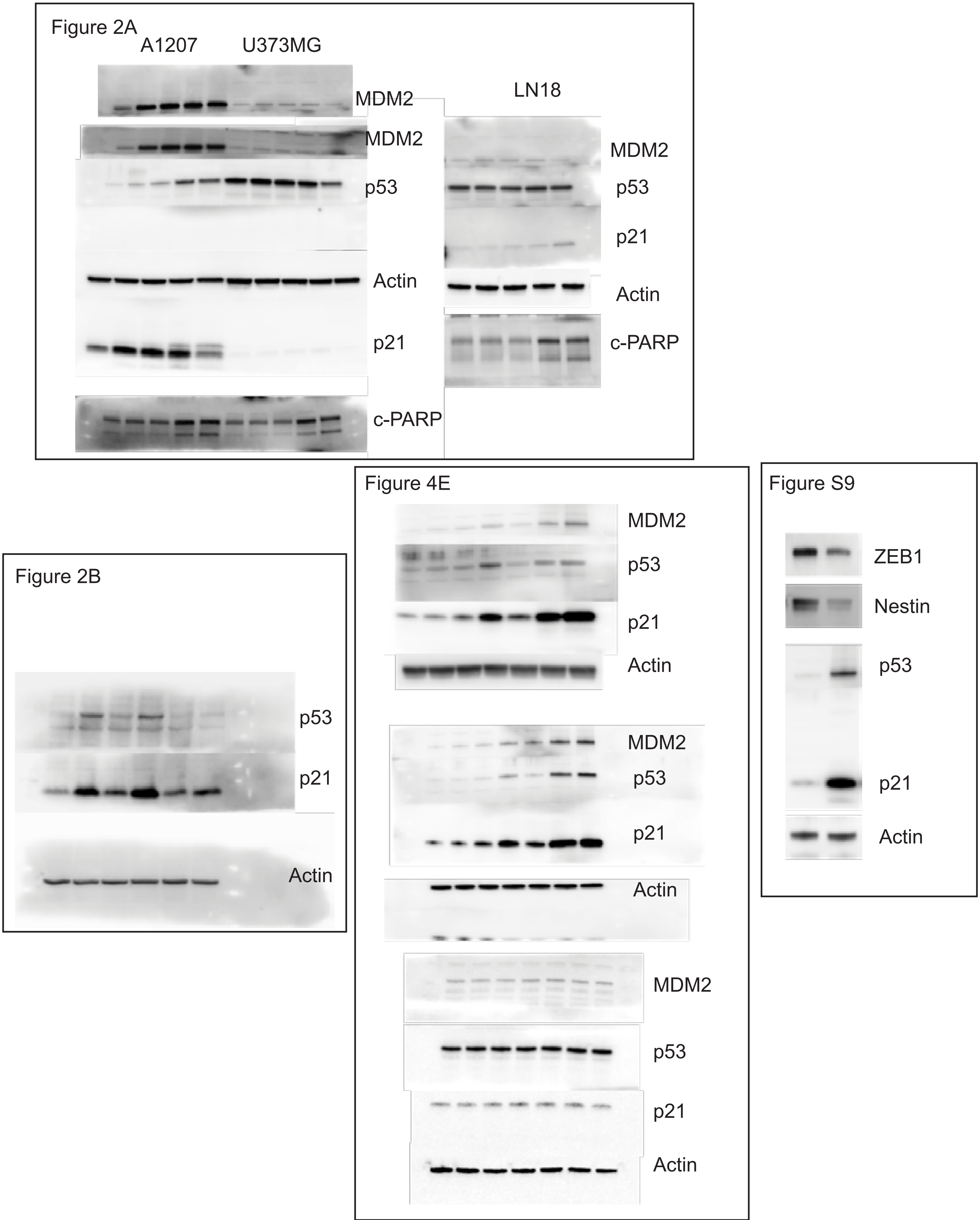

Supplement: Supplementary file 1 — Supplementary Figures [file 41419_2018_825_MOESM1_ESM.pdf]
